# Evaluation of Der p 10 in a Cohort of European Children: Role of Molecular Diagnostics and Clinical Features

**DOI:** 10.1155/2023/5551305

**Published:** 2023-06-19

**Authors:** Cristiana Indolfi, Giulio Dinardo, Angela Klain, Alessandra Salvatori, Marica Esposito, Viviana Vela, Fabio Decimo, Giorgio Ciprandi, Michele Miraglia del Giudice

**Affiliations:** ^1^Department of Woman, Child and General and Specialized Surgery, University of Campania “Luigi Vanvitelli”, Naples, Italy; ^2^Allergy Clinic, Casa di Cura Villa Montallegro, Genoa, Italy

## Abstract

**Background:**

Allergy toward the dust mite is steadily increasing on the European continent. This sensitization may be a risk factor for developing sensitization to other mite molecules such as tropomyosin Der p 10. This molecule often correlates with food allergy and the risk of anaphylaxis after ingesting mollusks and shrimps.

**Materials and Methods:**

We analyzed the sensitization profiles by ImmunoCAP ISAC of pediatric patients from 2017 to 2021. The patients under investigation were being followed for atopic disorders such as allergic asthma and food allergies. The study aimed to analyze the prevalence of sensitization toward Der p 10 in our pediatric population and assess the related clinical symptoms and reactions after ingestion of foods containing tropomyosins.

**Results:**

This study included 253 patients; 53% were sensitized toward Der p 1 and Der p 2; 10.4% were also sensitized to Der p 10. Assessing patients sensitized to Der p 1 or Der p 2, and Der p 10, we observed that 78.6% were affected by asthma (*p* < 0.005) and had a history of prior anaphylaxis after ingestion of shrimp or shellfish (*p* < 0.0001).

**Conclusion:**

The component-resolved diagnosis gave us a deeper understanding of patients' molecular sensitization profiles. Our study showed that a fair proportion of children sensitive to Der p 1 or Der p 2 are also sensitive to Der p 10. However, many patients sensitized to all three molecules had a high risk of asthma and anaphylaxis. Therefore, the assessment of Der p 10 sensitization should be considered in atopic patients with sensitization to Der p 1 and Der p 2 to avoid encountering possible adverse reactions after ingesting foods containing tropomyosins.

## 1. Introduction

House dust mite (HDM) is one of the most common indoor allergens. More than 50% of allergic patients and more than 80% of asthmatic children are sensitized to dust mites [1]. HDMs, especially *Dermatophagoides pteronyssinus* (DP), are considered an important source of allergic sensitization, and they are the main risk factor for allergic respiratory diseases in genetically predisposed patients [2, 3]. Der p 1 and Der p 2 are considered the major allergens of DP, as more than 90% of the mite-sensitized patients are positive for them [4]. Another novel HDM major allergen is Der p 23, with a reported incidence of 74% in individuals with DP sensitization [5]. Recent research suggests that Der p 23 is a major allergen already clinically significant in the first years of life [6, 7]. Furthermore, in HDM-allergic individuals, Der p 23 appears strongly related to asthma, allergic rhinitis, and atopic dermatitis in infancy [8, 9]. These findings support the inclusion of Der p 23-IgEs molecular testing in clinical HDM allergy suspicion.

**Figure 1:**
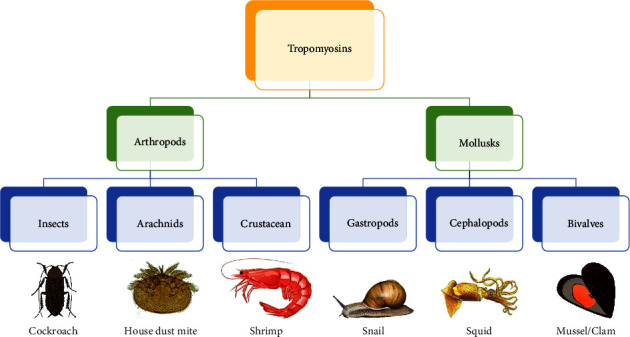
The family of tropomyosins.

Der p 10, on the other hand, is one of the minor allergens of HDM, with a reported prevalence among DP-sensitized patients between 5% and 18% [10, 11]. Der p 10 is a tropomyosin, one of the primary thermostable allergenic components responsible for the cross-reactivity across crustaceans, mites, insects, and nematodes [12] (Figure 1). It is regarded as the main invertebrate panallergen that sensitizes susceptible individuals by inhalation or ingestion [13].

In particular, Der p 10 shares high sequence homology with Pen a 1 (shrimp tropomyosin allergen), with an aminoacidic sequence similarity of 81% between shrimp and HDM tropomyosins and four identical IgE-binding epitopes [14, 15]. Shellfish is one of the leading causes of persisting throughout life food allergy and is a common cause of food-induced anaphylaxis [16]. Shellfish allergy affects up to 10% of the general population, especially in the Asia-Pacific regions [17–19]. In Western countries, children's self-reported rates of shellfish allergy range from 0.06% to 2%, but the actual prevalence in the general and pediatric population is underestimated [20]. Few studies investigated the clinical and biomolecular role of tropomyosins in HDM-sensitised children. The objective of our study was to evaluate the prevalence of sensitization toward Der p 10 in our pediatric population assessing the associated clinical symptoms and the incidence of allergic reactions after ingesting foods containing tropomyosins.

## 2. Materials and Methods

### 2.1. Patients

The study included consecutive children attending the Allergy and Pneumology Unit of Pediatric Clinic University of Campania “Luigi Vanvitelli” from 2017 to 2021. All patients aged between 1 and 18 years were followed for atopic disorders such as allergic asthma, atopic dermatitis, urticaria, allergic rhinitis, and food allergies and performed an ImmunoCAP ISAC.

### 2.2. Study Design

We analyzed the serum-specific IgE of molecules Der p 1, Der p 2, and Der p 10 retrospectively, and Der p 23 using the microarray method (ImmunoCAP ISAC, ThermoFisher Scientific, Milan, Italy). Molecular sensitization profiles obtained by ImmunoCAP ISAC were evaluated and compared with each other to assess possible cross-reactivity and correlations. Sensitization was defined when the value was higher than 0.3 ISU-E. The study considered patients' clinical data, such as asthma, atopic dermatitis, rhinitis, urticaria, and history of anaphylaxis after food ingestion. The molecular sensitization to Der p 1, Der p 2, Der p 10, and Der p 23 were compared to clinical data to assess differences between sensitized and not-sensitized populations. We evaluated four groups of patients: the first group was sensitized to Der p 1 or Der p 2, the second group was sensitized to Der p 10, the third group was sensitized to both Der p 10 and Der p 1 or Der p 2, and the fourth group was sensitized to both Der p 23 and Der p 1 or Der p 2.

### 2.3. Endpoint

The primary endpoint assessed the sensitization profile toward the Der p 1-Der p 2 and Der p 10, and Der p 23 molecules and the clinical and laboratory characteristics of sensitized patients. The secondary endpoint was to compare sensitized populations and to evaluate how sensitization to Der p 10 or Der p 23 can affect clinical manifestations in patients.

### 2.4. Statistical Analysis

Patients' characteristics were defined using descriptive statistics and expressed as a percentage. We used the *χ*^2^ test to compare the data obtained on the clinical and molecular sensitization profiles analyzed during the study. Significance was set for *p*-values < 0.05. All the analyses were performed using Microsoft Excel for Microsoft 365, Microsoft Inc., Redmond, Washington, USA, and IBM SPSS Statistics for Windows, Version 26.0. Armonk, NY: IBM Corp.

## 3. Results

The current study assessed the sensitization to Der p 1, Der p 2, and Der p 10 in 253 consecutive Caucasian children, 167 males and 86 females, ranging in age from 1 to 18 years, between 2017 and 2021. Furthermore, within this group of 253 children, we evaluated sensitization data regarding Der p 23 in a subset of 70 patients, 48 males and 22 females.

**Figure 2:**
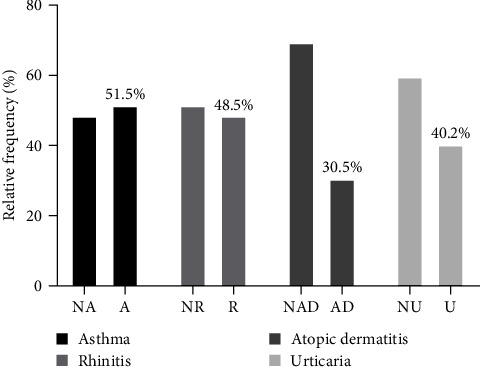
Clinical characteristics in patients sensitized to Der p 1–Der p 2. NA, nonasthmatics; A, asthamatics; NR, nonrhinitic; R, rhinitic; NAD, nondermatitic atopic; AD, atopic dermatitis; NU, nonhorticarial; U, horticarial.

The data analyses found that 53% of patients were sensitized to Der p 1 or Der p 2 and 9.1% to Der p 10. About patients' clinical data, 44.7% suffered from asthma, 32% from atopic dermatitis, 41.5% from urticaria, and 4.9% from rhinitis (Table 1). Data analysis of the first group of patients sensitized to Der p 1 or Der p 2 showed that patients with asthma were 51.5%, patients with atopic dermatitis were 30.6%, patients with urticaria were 40.3%, and patients with rhinitis were 48.5% (Figure 2). The 10.4% of patients sensitized to Der p 1 and Der p 2 were also sensitized to Der p 10, and the 54.5% of patients sensitized to Der p 1 and Der p 2 were also sensitized to Der p 23 (Table 2).

**Figure 3:**
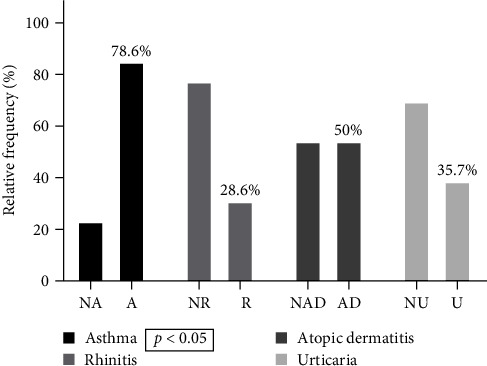
Clinical characteristics in patients sensitized to Der p 1–Der p 2–Der p 10. NA, nonasthmatics; A, asthamatics; NR, nonrhinitic; R, rhinitic; NAD, nondermatitic atopic; AD, atopic dermatitis; NU, nonhorticarial; U, horticarial.

Data analysis of the second group of patients sensitized to Der p 10 showed that patients with asthma were 73.9% (*p* < 0.01), patients with atopic dermatitis were 52.2%, patients with urticaria were 39.1%, and patients with rhinitis were 39.1%. The 89.6% (*p* < 0.0001) of patients sensitized to Der p 10 reported anaphylactic reactions after ingestion of shrimp or shellfish (Table 3). Data analysis of patients sensitized to Der p 23 showed that patients with asthma were 66.7% (*p* < 0.05), patients with atopic dermatitis were 19.0%, patients with urticaria were 42.9%, and patients with rhinitis were 57.1% (Table 4). Data analysis of the third group of patients sensitized to Der p 10 and Der p 1 or Der p 2 revealed that patients with allergic asthma were 78.6% (*p* < 0.05), patients with atopic dermatitis were 50%, patients with urticaria were 35.7%, and patients with allergic rhinitis were 28.6% (Figure 3). Der p 1 or Der p 2 sensitization was present in 61.9% of individuals who had previously had anaphylaxis to shrimp or shellfish.

**Figure 4:**
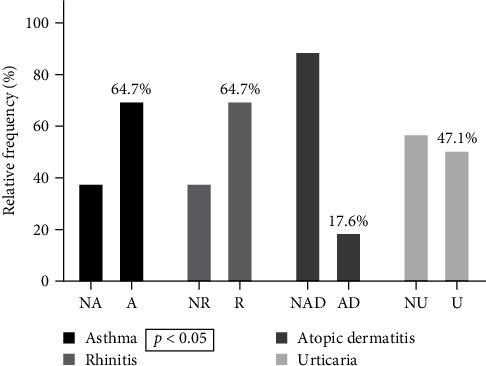
Clinical characteristics in patients sensitized to Der p 1–Der p 2–Der p 23. NA, nonasthmatics; A, asthmatics; NR, nonrhinitic; R, rhinitic; NAD, nondermatitic atopic; AD, atopic dermatitis; NU, nonhorticarial; U, horticarial.

Data analysis of the fourth group of patients sensitized to Der p 23 and Der p 1 or Der p 2 revealed that patients with allergic asthma were 64.7% (*p* < 0.05), patients with atopic dermatitis were 17.6%, patients with urticaria were 47.1%, and patients with allergic rhinitis were 64.7% (Figure 4). We performed the *χ*^2^ test regarding sensitization to Der p 10 and anaphylaxis after ingestion of shrimp and shellfish and Der p 10 and asthma, obtaining statistical significance (*p* < 0.0001 and *p* < 0.01, respectively) (Table 5).

## 4. Discussion

In our study, we evaluated 253 pediatric patients. More than half of the patients showed sensitization toward HDM, and most were affected by atopic disorders such as asthma and rhinitis. In literature, the prevalence of sensitization to Der p 10 is about 9%–18% in Europe and 5.6% in Spain [21, 22]. Our data analysis showed similar results, 9.1% of patients were sensitized to Der p 10, and 89.6% of these patients reported anaphylactic reactions after shrimp or shellfish ingestion (*p* < 0.0001). In comparison, 73.9% were affected by asthma (*p* < 0.01), with statistically significant results. In epidemiological research with 48 patients allergic to shellfish, 82% of them appeared to be sensitized to HDM [23]; in our analysis, 61.9% of patients allergic to shellfish were also sensitized to HDM. In our study, the Der p 10 sensitized group suffered more from atopic conditions such as asthma, rhinitis, and atopic dermatitis than Der p 1 and Der p 2 sensitized patients.

Regarding sensitization against Der p 23, we observed that 66.7% of patients were affected by asthma (*p* < 0.05). Based on our experience, we have observed that sensitization to multiple dust mite molecules is associated with a higher proportion of individuals with asthma among our patients. According to some studies, in HDM-allergic individuals, the likelihood of developing asthma is influenced by the number of allergen sources other than HDM [8] and the number of mite allergen molecules a person has become sensitized [24]. The importance of HDM sensitization is now taken into greater attention. It has been proposed that the primary sensitizer for shellfish allergies is inhalant exposure to HDM tropomyosin by subsequent IgE cross-reactivity with shellfish tropomyosin, an explanation for the later age of onset and prevalence of oral symptoms seen in the Asia-Pacific area, where HDM is highly common [20]. In general, it has been reported that in shellfish-sensitized children, the prevalence of HDM sensitization is high (∼90% in the Jirapongsananuruk study, ∼73% in the Chiang trial) [25, 26]. A Spanish study also supports these data by investigating patients with HDM and shrimp allergies. The authors found an almost complete inhibition of shrimp extract by a mite (*Chortoglyphus arcuatus*) in immunoblot inhibition studies, suggesting that HDMs are the primary sensitizers [20, 27]. Shrimp allergy in the Mediterranean is strictly associated with and almost always dependent upon HDM sensitization [23]. So far, it has focused on the association between Der p 10 and other tropomyosins as risk factors for shellfish and shrimp allergy. In our experience, we observed that about 10% of patients sensitized to HDM were also sensitized to Der p 10. The association between simultaneous sensitization to HDM and Der p 10 and anaphylaxis was statistically significant (*p* < 0.0001), and of this group of patients, 78.6% were asthmatic (*p* < 0.05). In Farioli et al. [28] study, including patients with reported reactions to shrimp, the authors found that the simultaneous positivity of all HDM recombinant sIgE allergens (nDer p 1, rDer p 2, and rDer p 10) corresponded to a 4.8% increase in the odds of developing shrimp allergy. Interestingly, the presence of asthma was associated with a 736% increase in the odds of developing symptoms after shrimp ingestion (Wald test: *p* = 0.002), with a 4.050% increase in the odds of developing asthma (Wald test: *p* < 0.0005) when positivity of anti-nDer p 1, 2, and 10 (*p* = 0.085) IgE levels were considered as single variable [28]. Concerning this finding, we think that patients, in particular asthmatic, sensitized exclusively to Der p 1 and 2 should be followed over time and repeat *in vivo* and *in vitro* tests (component resolved diagnosis (CRD) to check for sensitization to Der p 10 and other tropomyosins, which are the main responsible for the cross-reactivity between mollusks and anthropoids.

## 5. Conclusions

Our research confirms that dust mite allergy is a common condition in children. Few studies investigate sensitization to seafood and shrimp in children with mite allergies. In our research, we found that not only is Der p 10 statistically associated with anaphylaxis after ingestion of crustaceans and shrimp, but also that sensitization to Der p 10 in children sensitized to Der p 1 or Der p 2 is not uncommon. As evidenced by other studies, the sensitization to multiple dust mite molecules is linked to a higher prevalence of asthma among individuals. In conclusion, in children sensitized to HDM, it is essential to investigate a history of clinical reactions toward crustaceans and mollusks and possibly test for their sensitization *in vivo* and *in vitro* tests. Additionally, in our experience, the use of CRD would be helpful in identifying children who are sensitized to Der p 1, 2, and 10 not only in the context of atopic disorders but also as a risk factor for primary sensitization to crustaceans and shrimp, to prevent severe reactions and anaphylaxis.

## Figures and Tables

**Table 1:** Clinical characteristics and sensitization to Der p 10 and Der p 1 or Der p 2 of the whole population under study.

	Der p 1/Der p 2 sensitized	Der p 10 sensitized	Asthmatics	Atopic dermatitis	Urticaria	Rhinitis
Total patients	134 (53%)	23 (9.1%)	113 (44.7%)	81 (32%)	105 (41.5%)	111 (43.9%)

**Table 2:** Clinical characteristics and sensitization to Der p 10 of the population sensitized to Der p 1 or Der p 2.

	Der p 10	Der p 23	Asthmatics	Atopic dermatitis	Urticaria	Rhinitis
Patients Der p 1/2 sensitized	14 (10.4%)	18 (54.5%)	69 (51.5%)	41 (30.6%)	54 (40.3%)	65 (48.5%)
Patients Der p 1/2 not sensitized	9 (7.6%)	2 (5.7%)	44 (37%)	40 (33.6%)	51 (42.9%)	46 (38.7%)

**Table 3:** Clinical characteristics of the sensitized population in Der p 10.

	Asthmatics	Atopic dermatitis	Urticaria	Rhinitics	Anaphylaxis
Der p 10 sensitized	17 (73.9%); *p* < 0.01	12 (52.2%)	9 (39.1%)	9 (39.1%)	19 (89.6%); *p* < 0.0001
Der p 10 not sensitized	96 (41.7%)	69 (30%)	96 (41.7%)	102 (44.3%)	2 (0.9%)

**Table 4:** Clinical characteristics of the sensitized population in Der p 23.

	Asthmatics	Atopic dermatitis	Urticaria	Rhinitics	Anaphylaxis
Der p 23 sensitized	14 (66.7%); *p* < 0.05	4 (19.0%)	9 (42.9%)	12 (57.1%)	2 (9.5%)
Der p 23 not sensitized	19 (38.8%)	18 (36.7%)	25 (51.0%)	12 (24.5%)	0 (0.0%)

**Table 5:** Comparison of clinical characteristics of the population sensitized to Der p 10 and Der p 23 and Der p 1 or Der p 2.

	Asthmatics	Atopic dermatitis	Urticaria	Rhinitics	Anaphylaxis
Patients Der p1/2 sensitized	14 (10.4%)	54 (40.3%)	69 (51.5%)	41 (30.6%)	13 (9.7%)
Der p 10 sensitized	17 (73.9%); *p* < 0.01	12 (52.2%)	9 (39.1%)	9 (39.1%)	19 (89.6%); *p* < 0.0001
Der p 23 sensitized	14 (66.7%); *p* < 0.05	4 (19%)	9 (42.9%)	12 (57.1%)	2 (9.5%)
Der p 1-2-10 sensitized	11 (78.6%); *p* < 0.05	7 (50%)	5 (35.7%)	4 (28.6%)	11 (78.6%); *p* < 0.0001
Der p 1-2-23 sensitized	11 (64.7%); *p* < 0.05	3 (17.6%)	8 (47.1%)	11 (64.7%)	2 (11.8%)

## Data Availability

Data are available from the corresponding author upon reasonable request.
